# Study on the Multi-Stage Evolution of Thermal Runaway and the Flammability Threshold of Gas Generation in Lithium Iron Phosphate Batteries Based on SOC Gradient

**DOI:** 10.3390/mi16050544

**Published:** 2025-04-30

**Authors:** Changbao Qi, Hewu Wang, Minghai Li, Cheng Li, Yalun Li, Ningning Wei, Huipeng Zhang

**Affiliations:** 1 College of Zhan Tianyou, Dalian Jiaotong University, Dalian 116028, China; 1707@sdp.edu.cn (C.Q.);; 2 State Key Laboratory of Intelligent Green Vehicle and Mobility, Tsinghua University, Beijing 100084, China; 3 Department of Mechanical and Electronic Engineering, Yuncheng University, Yuncheng 044000, China

**Keywords:** lithium iron phosphate batteries, different SOC, gas generation during thermal runaway, flammability limits

## Abstract

Lithium batteries are widely used in fields such as engineering micro-machines, robotics, and transportation. However, safety issues caused by battery thermal runaway limit their further promotion. This study used a sealed heating pressure chamber (SHPC) to perform “heat-wait-seek (HWS)” stepwise heating on a 50 Ah lithium iron phosphate (LiFePO_4_) battery to trigger thermal runaway. It was found that the state of charge (SOC) has a significant impact on the safety of the battery. There was no significant correlation between the valve opening temperature (T_1_) and the temperature at which the battery’s thermal runaway rapidly self-heats (T_2_) and SOC. However, as SOC increased, the maximum temperature (T_3_) of the battery’s thermal runaway increased, reaching up to 357.4 °C. The mass loss rate due to thermal runaway increased with SOC. The critical point of the battery’s safety valve was essentially independent of SOC and was mainly influenced by temperature. After thermal runaway, the mixed gas was passed through a gas chromatograph (GC) to detect its composition. When the SOC was below 50%, the total gas production from thermal runaway increased slowly (0.68–0.90 mol). Above 50% SOC, the total gas production from the battery increased sharply (at 75% SOC, 1.17504 mol; at 100% SOC, 2.33047 mol). Among these gases, the amount of H_2_ increased sharply with SOC (from 0.01 mol at 0% SOC to 0.93 mol at 100% SOC), while the amount of CO_2_ remained almost constant. Considering the inerting effect of CO_2_ in the gas produced during thermal runaway of LiFePO_4_ batteries, the lower flammability limit of the mixed gas increased as SOC decreased (from 6.91% at 100% SOC to 55.43% at 0% SOC). The risk of explosion during thermal runaway of high SOC batteries significantly increased. Notably, within the SOC range of 25% to 100%, the flammable range remained stable at 34–43%, but at 0% SOC, it sharply dropped to 0.5%. Therefore, batteries that are deeply discharged have higher safety.

## 1. Introduction

With the mechanical industry shifting towards green and intelligent transformation, lithium batteries are widely used in fields such as industrial robots, drones, construction machinery, vehicles, and marine vessels due to their high energy density and long lifespan. However, the issue of lithium battery thermal runaway [1] has limited its further application due to uncontrolled temperature rise, flammable gas eruption, and explosions [2,3,4].

Thermal runaway is typically triggered by various factors such as mechanical abuse [5,6,7], high temperatures [8,9], internal short circuits [6,10,11], and overcharging [12,13]. Once thermal runaway occurs, a large amount of heat is generated inside the battery, causing the SEI film to melt, the positive and negative electrodes to decompose, and the electrolyte to undergo intense and complex chemical reactions with the electrodes, producing large amounts of flammable gases and CO_2_ [14]. As the mixed gases accumulate, the safety valve opens, and large amounts of flammable gas, electrolyte vapor, and CO_2_ rapidly escape from the safety valve [15]. The mixture of flammable gases and electrolyte vapors with oxygen in the air forms a highly combustible and explosive gas cloud, significantly threatening the safety of equipment and the environment [16,17,18].

Currently, there are many studies on battery thermal runaway behavior and the gas generation during thermal runaway. Qingsong Zhang et al. [19] triggered the thermal runaway of 18,650 commercial lithium batteries using surface heating and employed Raman spectroscopy to measure the composition of the thermal runaway gases. After the safety valve opened during thermal runaway, the main gases detected were CO, CO_2_, H_2_, CH_4_, C_2_H_4_, and C_3_H_6_. Wei et al. [17] conducted thermal runaway testing on commercial lithium iron phosphate (LiFePO_4_) and ternary lithium batteries using an adiabatic calorimeter and transferred the gases generated during thermal runaway to a gas chromatograph for component analysis. They found that both ternary lithium batteries and LiFePO_4_ batteries produced gases including CO, CO_2_, and hydrocarbons, but the LiFePO_4_ battery generated more H_2_, thus having a lower explosion limit. Qi et al. [20] tested ternary lithium batteries at different SOC states and found that when the battery was below 50% SOC, the hazards caused by thermal runaway were smaller, with the main gases generated being CO_2_, H_2_, and CO. Cui et al. [21] conducted thermal runaway experiments on ternary lithium and LiFePO_4_ batteries, discovering that the main gases generated during thermal runaway for both types of batteries were H_2_, CH_4_, and C_2_H_4_.

Previous studies mainly focused on small-capacity LiFePO_4_ batteries’ adiabatic thermal runaway gas generation or thermal runaway triggered by lateral heating. However, there are fewer studies on large-capacity batteries triggered by stepwise uniform heating in an environmental chamber and the subsequent gas composition analysis. This study uses a 50 Ah LiFePO_4_ battery as the research object, with SOCs set at 0%, 25%, 50%, 75%, and 100%. The initial temperature is 30 °C, and the humidity is 0 (air is replaced with pure nitrogen). A 5 °C heating step is used, and the experiments are conducted using a self-made sealed heating pressure chamber (SHPC) to trigger thermal runaway. The gas generated after thermal runaway is transferred to a gas chromatograph (GC) for composition analysis. The amount of each gas component in the mixed gases is calculated based on the pressure data from the sealed heating pressure chamber (SHPC). The inerting effect of CO_2_ in the mixed gases is considered, and the flammability limit of the mixed gases is calculated. This study is of great significance for evaluating the critical temperature of thermal runaway in lithium iron phosphate batteries under different states of charge (SOC) and for guiding the thermal runaway design of both individual cells and battery modules. In addition, the investigation into gas generation and explosion risk during thermal runaway provides important guidance for developing control strategies that combine temperature threshold signals to rapidly reduce SOC, thereby effectively controlling the heat generation, gas release, and explosion risk associated with battery thermal runaway.

## 2. Experimental Methodology

### 2.1. Battery Samples

In this study, a commercial prismatic battery with a rated capacity of 50 Ah was used for the experiments. The battery’s cathode material is LiFePO_4_, the anode material is graphite, and the electrolyte is in liquid form. The dimensions of the battery are 148 × 95.4 × 39.7 mm. To minimize experimental variability, the same batch of batteries was used in this experiment, and five battery samples with nearly identical internal resistance (0.5 mΩ) were selected.

According to the battery parameters in Table 1, the batteries were charged to 3.65 V using a constant voltage current (CC) method and then discharged to 2.5 V in constant voltage mode (CV) with a cutoff current of 1/20 C, conducting three charge-discharge cycles. The initial capacity of the battery was measured to be 50 Ah, and the tested batteries were set to five different SOCs (100%, 75%, 50%, 25%, 0%). Other battery parameters can be found in Table 1.

### 2.2. Experimental Equipment

The main equipment used in this study includes the charge-discharge testing equipment, a sealed heating pressure chamber (SHPC) (Tsinghua University, China), and a gas chromatograph (GC).

The charge-discharge equipment used is the NEWARE CT-4008-5V100A-NTA (manufactured by NEWARE Company in Guangzhou, China), with a data recording frequency of 10 Hz and a response time of no more than 10 ms.

**Figure 1:**
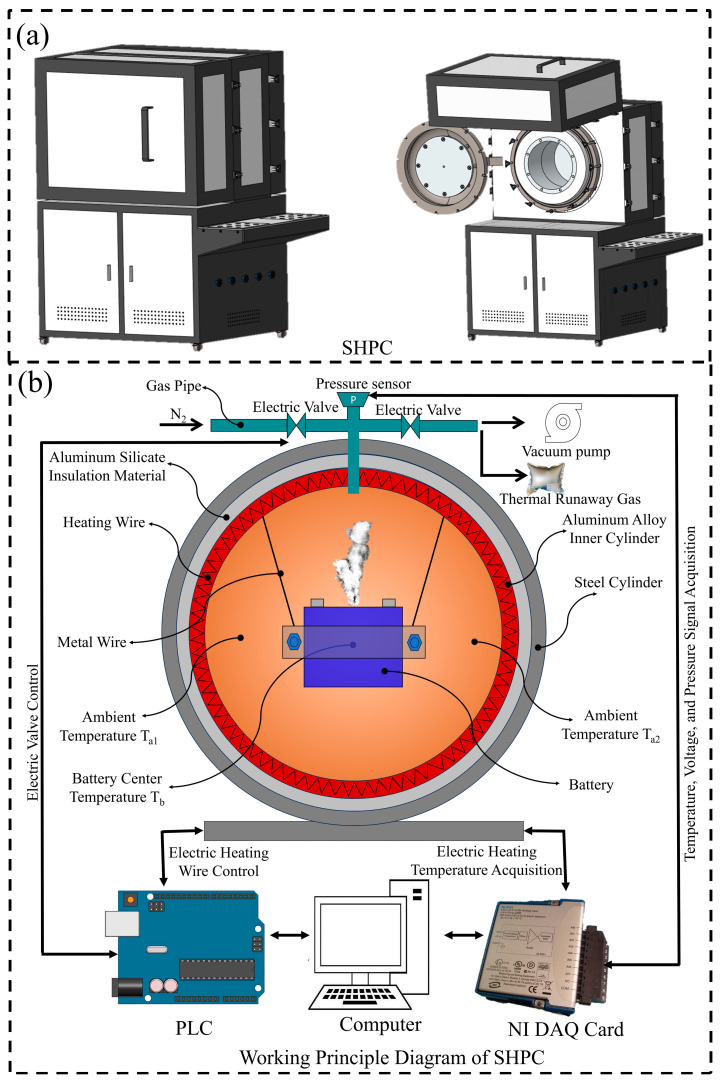
Working principle diagram of the sealed heating pressure chamber (SHPC). (**a**) Appearance of the sealed heating pressure chamber (SHPC) equipment. (**b**) Working principle of the sealed heating pressure chamber (SHPC).

As shown in Figure 1a, the sealed heating pressure chamber (SHPC) (manufactured in China by Tsinghua University) consists of a sealed chamber, electric heating system, temperature and pressure signal acquisition system, and gas displacement system.

As shown in Figure 1b, the outermost layer of the sealed chamber is made of carbon steel, which serves as the pressure-bearing layer, with a maximum gas pressure capacity of 2 MPa. The inner layer is made of aluminum alloy, with a battery fixation structure inside. Between the inner and outer layers, aluminosilicate material is used for insulation.

The electric heating system has heating wires embedded in the inner layer (aluminum alloy) of the sealed chamber. The heating time and power are controlled by a PLC system through a computer program. The heating system raises the temperature of the chamber by heating the entire inner aluminum alloy layer and transferring the heat to the battery in the center of the chamber via the air.

A K-type thermocouple (range: 0–1300 °C) is installed on the chamber to collect battery temperature signals. A gas pressure sensor (range: 0–400 KPa) detects pressure changes inside the chamber, and voltage signal wires are used for battery voltage signal detection. The temperature, voltage, and chamber pressure signals are collected using a National Instruments (NI) data acquisition card.

The chamber is equipped with intake and exhaust electromagnetic valves. When the exhaust valve is opened and the intake valve is closed, the vacuum pump can evacuate gas from the chamber. When the exhaust valve is closed and the intake valve is opened, nitrogen gas (99.9% purity) can be injected into the chamber. After the experiment, the exhaust valve is opened, and the intake valve is closed to collect the thermal runaway gases using an aluminum foil gas bag.

The gas composition analysis device used is a gas chromatograph (GC) 1300_1310 (manufacturer: Thermo Fisher Scientific, origin: Singapore), equipped with a flame ionization detector (FID), electron capture detector (ECD), nitrogen-phosphorus detector (NPD), and thermal conductivity detector (TCD). The gas composition is detected by connecting the aluminum foil gas bag to the intake port.

**Figure 2:**
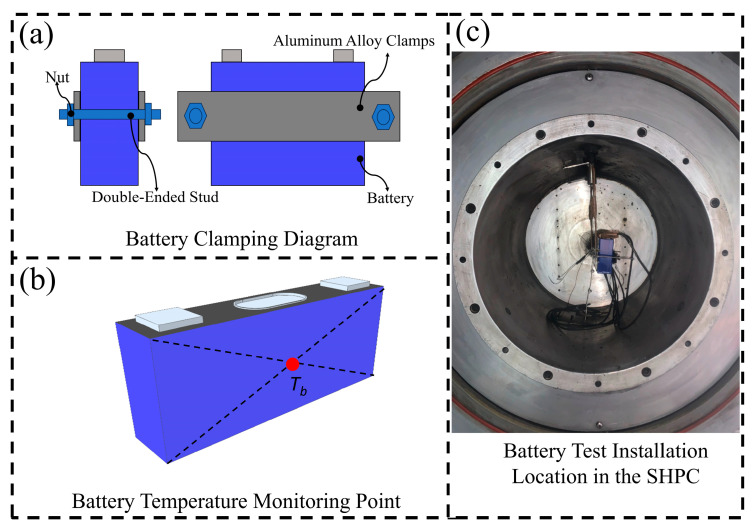
Battery clamping diagram. (**a**) Battery clamps. (**b**) Thermocouple detection position. (**c**) Battery installation position.

As shown in Figure 2a, two aluminum alloy clamps are used to tighten the large surface of the battery with bolts and nuts. As shown in Figure 2b, a thermocouple is attached to the center of the battery’s large surface and fixed by the aluminum alloy clamps. Figure 2c shows the position of the battery in the sealed heating pressure chamber (SHPC) equipment after being clamped.

### 2.3. Experimental Procedure

**Figure 3:**
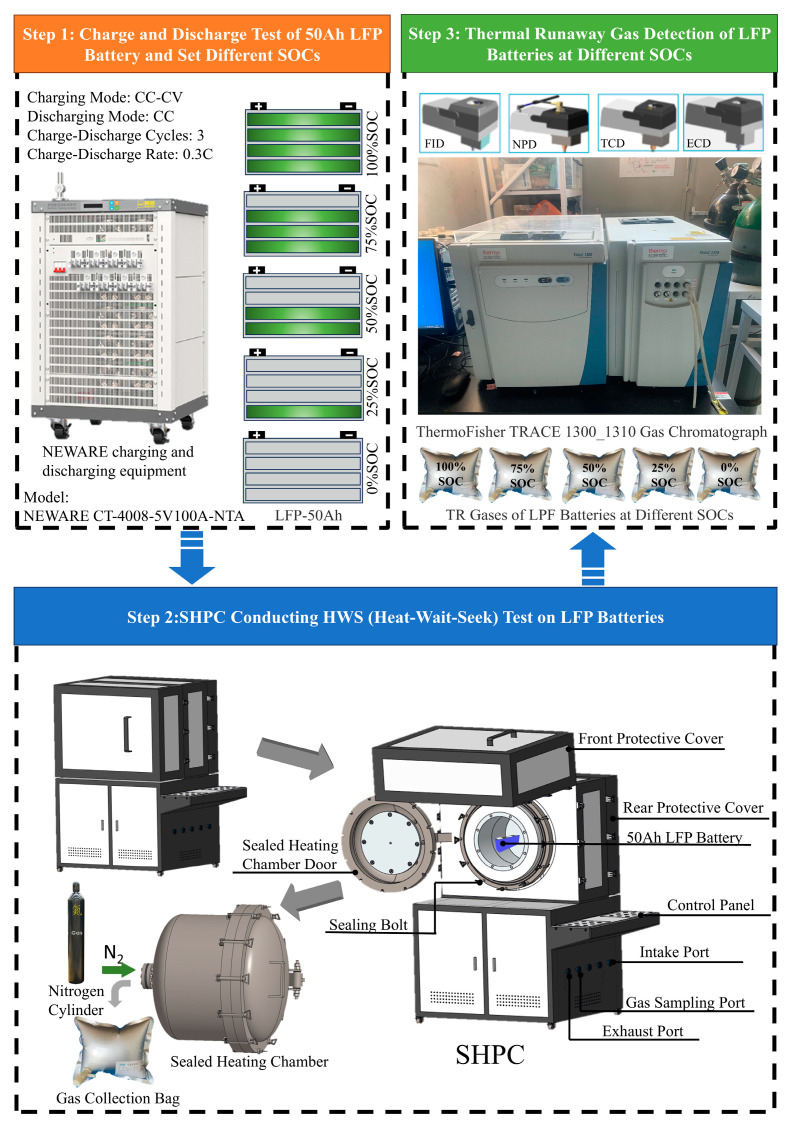
Experimental procedure.

**Figure 4:**
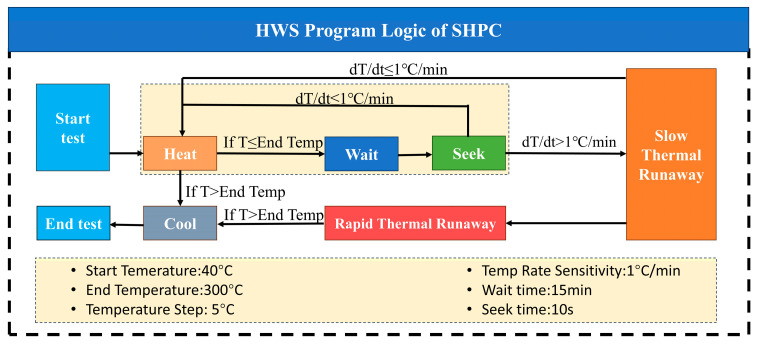
HWS program of the sealed heating pressure chamber (SHPC).

As shown in Figure 3, according to the specifications of the 50 Ah LFP battery, the battery was subjected to charge-discharge cycles at a rate of 0.3 C. The battery was then set to five different SOC levels: 100%, 75%, 50%, 25%, and 0%. The battery was placed into the sealed chamber of the sealed heating pressure chamber (SHPC) for fixation. The chamber door was closed, and the vacuum pump was turned on to evacuate the chamber to a relative pressure of −95 KPa. After the vacuum pump was turned off, nitrogen gas (99.9% purity) was injected into the chamber until the absolute pressure reached 0 KPa. This gas exchange process was repeated three times, reducing the oxygen content in the chamber to 0.0025%. When the electric heating system is activated, the equipment will heat according to the HWS control program shown in Figure 4. The “HWS” parameters are set in accordance with the specifications of the thermal management testing system for power batteries—accelerated calorimeter (EVARC) produced by the THT Company located in Staffordshire, UK. “H” represents the heating stage, with a temperature increase of 5 °C each time. “W” represents the waiting stage, with a waiting time of 15 min. “S” represents the search stage, with a search time of 10 s. The criterion for ending the search stage is when dT/dt ≤ 1 °C/min. If this condition is met, the HWS stage ends. This process is referred to as the stepwise heating process. The starting temperature in this process is 40 °C (the most extreme environmental temperature), and the step termination temperature is 300 °C (the thermal runaway triggering temperature of LiFePO_4_ batteries under different SOCs is all below 300 °C). During the heating process, the surface temperature, ambient temperature, absolute pressure in the chamber, and battery voltage signals are collected in real time. After the battery’s thermal runaway cooling, the thermal runaway gases are collected using aluminum foil gas bags. The thermal runaway gases from five different SOCs are injected into the GC device for the detection of gas composition during thermal runaway.

After the battery’s thermal runaway process cooled down, the thermal runaway gases were collected using an aluminum foil gas bag. The thermal runaway gases at the five different SOC levels were then injected into the GC device for component analysis of the thermal runaway gases.

## 3. Results

### 3.1. Battery TR Temperature and Voltage Changes

**Figure 5:**
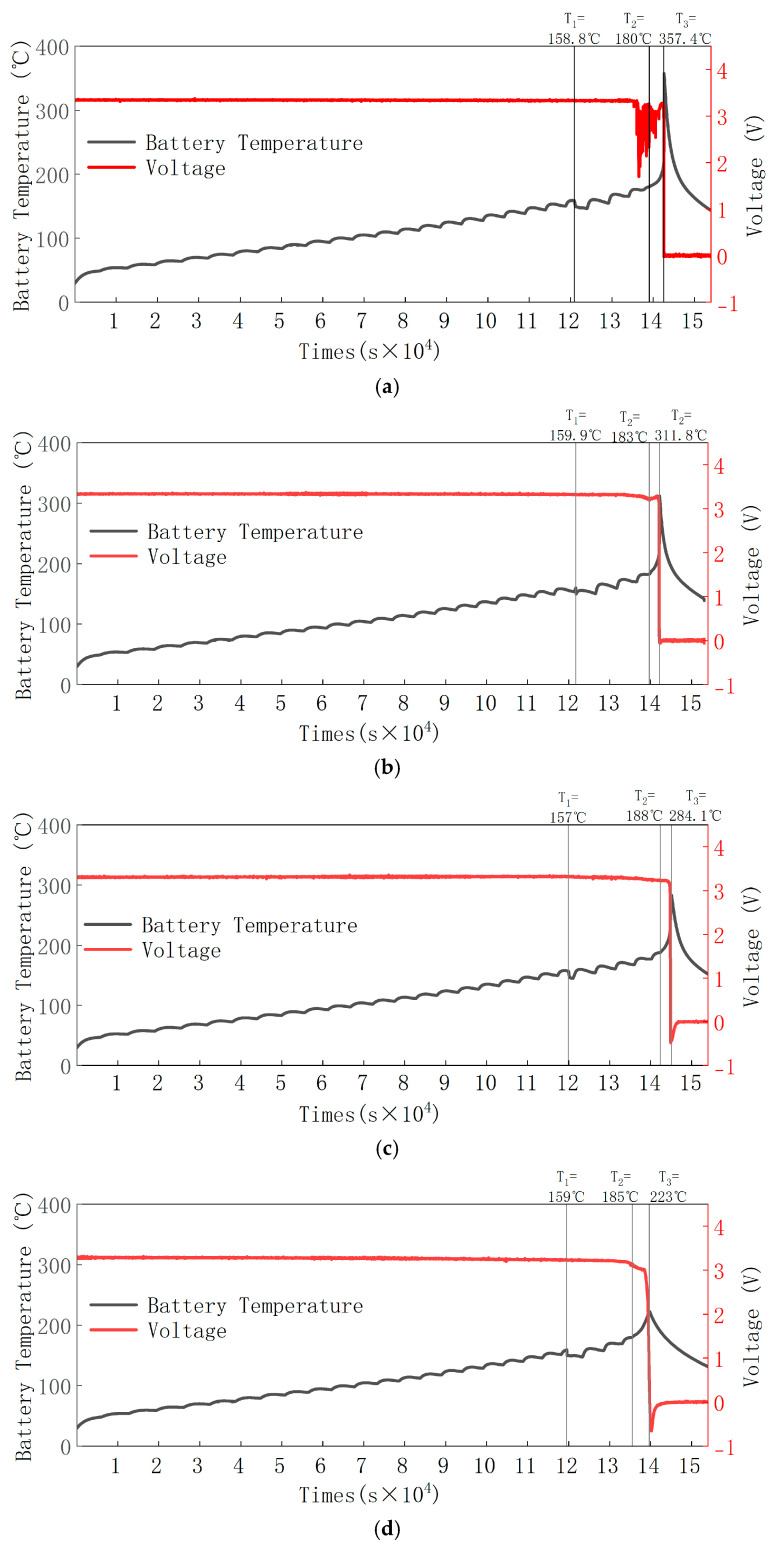

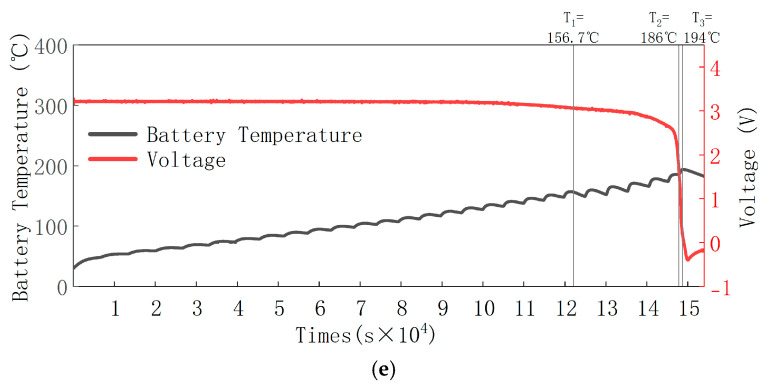
Thermal runaway temperature and voltage of LiFePO_4_ batteries at five different SOC levels. (**a**) Thermal runaway temperature and voltage of LiFePO_4_ battery at 100% SOC. (**b**) Thermal runaway temperature and voltage of LiFePO_4_ battery at 75% SOC. (**c**) Thermal runaway temperature and voltage of LiFePO_4_ battery at 50% SOC. (**d**) Thermal runaway temperature and voltage of LiFePO_4_ battery at 25% SOC. (**e**) Thermal runaway temperature and voltage of LiFePO_4_ battery at 0% SOC.

The sealed heating pressure chamber (SHPC) was used to perform “HWS” stepwise environmental heating on LiFePO_4_ batteries at five SOC levels (100%, 75%, 50%, 25%, 0%). Each heating phase was 5 °C, and the temperature of the battery’s large surface (referred to as the battery temperature) was measured using a thermocouple attached to the side of the battery. The voltage of the positive and negative electrodes was recorded via voltage lines. The thermal runaway process of the battery is shown in Figure 5, where T_1_ represents the valve opening temperature, T_2_ represents the rapid self-heating start temperature, and T_3_ represents the maximum temperature during thermal runaway [22].

Figure 5a shows the temperature parameters at the critical points of the thermal runaway process for the 100% SOC battery. The valve opening temperature (T_1_) is 158.8 °C, the rapid self-heating start temperature (T_2_) is 180 °C, and the maximum temperature during thermal runaway (T_3_) is 357.4 °C. The battery voltage does not show significant changes during the initial stage after the safety valve opens, and after 14,372 s, the voltage starts to fluctuate, continuing until the intense thermal runaway stage, at which point the voltage rapidly drops to 0 V.

Figure 5b shows the temperature parameters at the critical points of the thermal runaway process for the 75% SOC battery. The valve opening temperature (T_1_) is 159.9 °C, the rapid self-heating start temperature (T_2_) is 183 °C, and the maximum temperature during thermal runaway (T_3_) is 311.8 °C. The battery voltage remains relatively stable in the early stages after the safety valve opens, but after 12,519 s, the voltage starts to fluctuate, continuing until the intense thermal runaway stage, where the voltage drops sharply to 0 V.

Figure 5c shows the temperature parameters at the critical points of the thermal runaway process for the 50% SOC battery. The valve opening temperature (T_1_) is 157 °C, the rapid self-heating start temperature (T_2_) is 188 °C, and the maximum temperature during thermal runaway (T_3_) is 284.1 °C. The battery voltage initially does not change significantly after the safety valve opens, but after 17,166 s, the voltage starts to fluctuate, continuing until the intense thermal runaway stage, where the voltage rapidly drops to 0 V.

Figure 5d shows the temperature parameters at the critical points of the thermal runaway process for the 25% SOC battery. The valve opening temperature (T_1_) is 159 °C, the rapid self-heating start temperature (T_2_) is 185 °C, and the maximum temperature during thermal runaway (T_3_) is 223 °C. The battery voltage remains stable during the initial stage after the safety valve opens, and after 15,086 s, the voltage starts to fluctuate. It continues fluctuating until the intense thermal runaway stage, where the voltage rapidly drops to 0 V.

Figure 5e shows the temperature parameters at the critical points of the thermal runaway process for the 0% SOC battery. The valve opening temperature (T_1_) is 156.7 °C, the rapid self-heating start temperature (T_2_) is 186 °C, and the maximum temperature during thermal runaway (T_3_) is 194 °C. The battery voltage starts to slowly decrease after the safety valve opens, and around 186 °C, the voltage drops sharply to 0 V.

**Figure 6:**
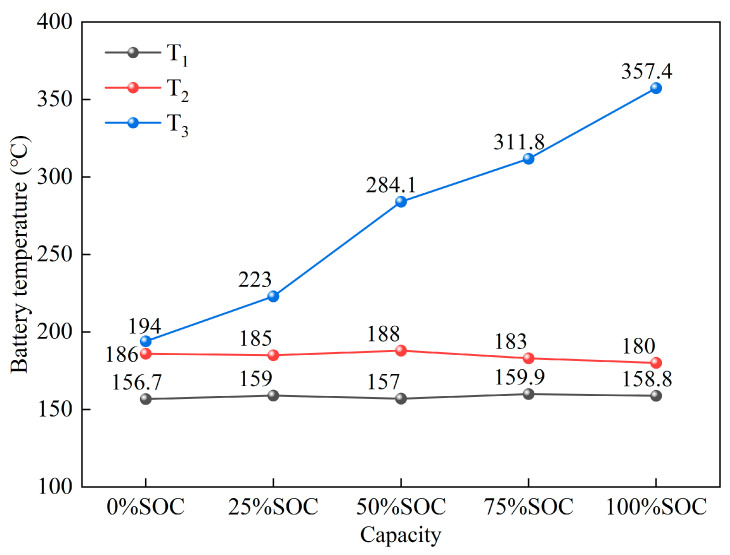
Critical thermal runaway temperatures of batteries at different SOC levels.

As shown in Figure 6, the valve opening temperature (T_1_) during the battery thermal runaway is in the range of 156.7 °C to 159.9 °C at different SOC levels, with only slight temperature fluctuations. This indicates that there is no significant correlation between the valve opening temperature (T_1_) and SOC, and it mainly depends on the vapor pressure of the electrolyte in the LiFePO_4_. As the temperature rises, the electrolyte gradually evaporates, and the internal pressure of the battery increases, eventually causing the safety valve to open.

The rapid self-heating start temperature (T_2_) during thermal runaway is in the range of 180 °C to 186 °C at different SOC levels, with minimal temperature change. This suggests that there is no significant correlation between the rapid self-heating start temperature (T_2_) and SOC, and it mainly depends on the intrinsic characteristics of the battery’s anode and cathode materials. The separator between the anode and cathode undergoes pyrolysis in a high-temperature environment, leading to a short circuit between the electrodes, which causes intense reactions between the anode, cathode, and electrolyte, triggering rapid thermal runaway and heat generation.

The maximum temperature during thermal runaway (T_3_) is positively correlated with SOC, meaning that a higher SOC leads to a higher maximum temperature (T_3_) during thermal runaway, reaching up to 357.4 °C. This phenomenon indicates that when the SOC is higher, the battery stores more energy. During thermal runaway, internal short circuits occur within the battery, and high-SOC batteries have higher short-circuit currents, generating more heat. Additionally, the chemical activity of both the anode and cathode materials increases. The decomposition reactions of the electrolyte, SEI film, redox reactions of the cathode material, de-lithiation reactions of the anode material, and other side reactions intensify with the high temperature, further raising the internal temperature of the battery. As a result, the maximum temperature (T_3_) during thermal runaway is higher in high-SOC batteries.

### 3.2. Mass Loss

**Figure 7:**
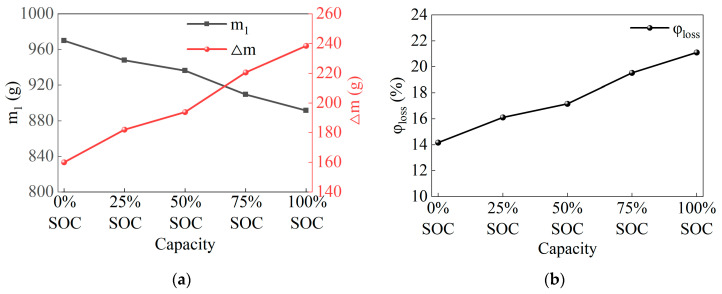
Mass loss before and after battery thermal runaway. (**a**) Remaining residue mass and mass loss after battery thermal runaway. (**b**) Battery thermal runaway mass loss rate.

As shown in Table 2, the battery mass and residue mass before and after thermal runaway were precisely measured using a milligram mass tester (manufacturer: KAIFENG Company, Jinhua City, China). Before the experiment, the initial mass of the battery (m_0_) was measured to be 1130 g. After the experiment, it was found that the residue mass after thermal runaway of the 100% SOC battery was the smallest, only 891.6 g, while the residue mass after thermal runaway of the 0% SOC battery was the largest, reaching 970.0 g. As seen in Figure 7a, the residue mass of the battery is inversely proportional to the SOC, meaning that the higher the SOC, the smaller the residue mass after thermal runaway. Meanwhile, the mass loss Δm of the battery is approximately directly proportional to the SOC, meaning that the higher the SOC, the greater the mass loss Δm.

To quantify the mass loss of different batteries [23], the mass loss rate φ_loss_ is defined as the standard for the mass loss before and after thermal runaway. The calculation formula is as follows:

(1) $$\varphi_{loss}=(m_{0}-m_{1})/m_{0}\times 100\%$$

where m_0_ is the initial mass (g), m_1_ is the post-experiment mass (g), and φ_loss_ is the mass loss rate (%).

As shown in Figure 7b, the mass loss rate is lowest at 14.16% for the 0% SOC battery, while the highest mass loss rate is 21.10% for the 100% SOC battery. The mass loss rates for 25% SOC (16.11%), 50% SOC (17.15%), and 75% SOC (19.52%) show that the mass loss rate is directly proportional to the SOC. This is because the higher the SOC, the higher the thermal runaway temperature, which leads to more intense chemical reactions inside the battery, causing more electrolyte and anode/cathode materials to be ejected. Further analysis reveals that the mass loss rate triggered by thermal runaway in a 50 Ah LiFePO_4_ battery using a stepwise heating method ranges from approximately 14% to 21%. This result indicates that SOC has a decisive effect on the thermal runaway mass loss rate.

### 3.3. Appearance Morphology Analysis

**Figure 8:**
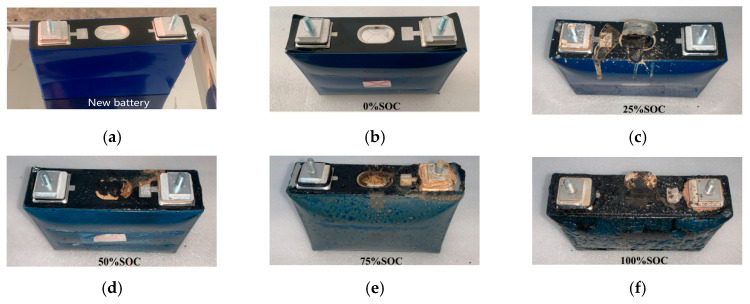
Appearance before and after battery thermal runaway. (**a**) Appearance of fresh battery before thermal runaway. (**b**) Appearance of 0% SOC battery after thermal runaway. (**c**) Appearance of 25% SOC battery after thermal runaway. (**d**) Appearance of 50% SOC battery after thermal runaway. (**e**) Appearance of 75% SOC battery after thermal runaway. (**f**) Appearance of 100% SOC battery after thermal runaway.

Figure 8a shows the fresh battery before the experiment, with a smooth and even surface and an intact safety valve.

Figure 8b indicates that the appearance of the 0% SOC battery after thermal runaway remains relatively intact, with the battery casing clean, showing no cracks or melting. However, due to the evaporation of the internal electrolyte, the internal pressure increases, causing the aluminum casing to expand and deform. The safety valve does not fully rupture but only partially cracks, with internal electrolyte vapor escaping through the crack. Because the battery is at 0% SOC, the electrolyte only partially evaporates, resulting in limited internal pressure increase, which causes the partial rupture of the safety valve and the release of some of the electrolyte.

Figure 8c shows that the appearance of the 25% SOC battery after thermal runaway remains relatively intact, but traces of electrolyte leakage are visible on the battery surface, and solid residue remains. The safety valve is fully opened. This is because the electrolyte evaporates due to heating, and side reactions inside the battery generate gases, increasing the internal pressure. As the internal pressure rises before the safety valve opens, the battery casing deforms. After the valve opens, the anode and cathode materials, along with the pyrolysis products, electrolyte vapor, and thermal runaway gases, are expelled from the battery.

Figure 8d indicates that the 50% SOC battery has a deformed casing after thermal runaway, with an oily solid residue covering the surface. The safety valve has fully opened. This is because after heating, the electrolyte inside the battery evaporates, and side reactions between the electrolyte and electrodes generate gas. The internal pressure rises quickly, causing the battery casing to deform and the safety valve to fully open. The electrolyte vapor and side reaction gases, along with small solid particles from inside the battery, are expelled, and after cooling, the gas cools and adheres to the surface of the battery, forming an oily solid substance.

Figure 8e indicates that after thermal runaway, the appearance of the 75% SOC battery also shows deformation of the casing, with the safety valve fully opened. The surface of the casing is covered by a thick layer of oily solid material. This is because after heating, the electrolyte inside the battery evaporates, and side reactions between the electrolyte and electrodes generate gas. The internal pressure of the battery rises rapidly, causing the casing to deform. The safety valve fully opens, and the electrolyte vapor and side reaction gases, along with small solid particles from inside the battery, are expelled. After cooling, the gas condenses and adheres to the surface of the battery, forming an oily solid substance. Since the battery is at 75% SOC, the side reactions generate more gas, the thermal runaway temperature is higher, and more electrolyte vapor is expelled, resulting in a thicker oily solid layer.

Figure 8f shows that after thermal runaway, the 100% SOC battery remains undamaged and unmelted, but the safety valve is fully opened. The surface of the battery is covered by a thick layer of oily solid material (which is a byproduct of side reactions), with black solid residues attached. This is because after heating, the electrolyte inside the battery evaporates, raising the internal pressure. Simultaneously, side reactions generate more gas, further increasing the internal pressure. When the critical point for the safety valve’s rupture is reached, the valve fully ruptures, and the high-pressure internal gas expels small amounts of anode and cathode materials along with the SEI film. As the battery continues to heat up, the SEI film decomposes, and intense chemical reactions occur between the anode, cathode, and residual electrolyte, triggering secondary ejection. More solid materials and liquid vapor are expelled from the battery through the safety valve. After thermal runaway, the vapor condenses into an oily solid, which adheres to the surface of the battery. Since the battery is at 100% SOC and is in a fully charged state, more liquid electrolyte is expelled, and the oily solid layer is thicker after cooling. “Black solid residue” is caused by the jetting resulting from severe thermal runaway of the battery at 100% SOC, during which graphite material from the battery’s anode is expelled to the outside of the battery casing.

In summary, for the 50 Ah LiFePO_4_ battery undergoing thermal runaway at five different SOC levels, the casing of the battery underwent consistent deformation, but there was no rupture or melting. Because the maximum temperature during thermal runaway was only 357.4 °C, which did not reach the melting point of the aluminum casing (660 °C), the casing remained intact. The safety valve experiences partial rupture at 0% SOC and rupture at 25% SOC, 50% SOC, 75% SOC, and 100% SOC. The opening temperature of the safety valve, as shown in Figure 6, is around 158 °C (ranging from 156.7 °C to 159.9 °C). This indicates that the critical point for the battery safety valve is essentially independent of the SOC and is primarily influenced by temperature. At 0% SOC, due to the limited energy inside the battery, the jet pressure during secondary ejection is low, and the safety valve is not fully breached, resulting only in a small pressure relief hole. However, at SOC levels greater than 25%, as the battery’s energy increases and thermal runaway intensifies, the pressure from the secondary ejection jet rises, fully breaching the safety valve. This indicates that when the battery capacity exceeds 25% SOC, thermal runaway will lead to the complete opening of the safety valve. Based on the oily solid material attached to the battery surface, it can be seen that the amount of liquid expelled from the battery is directly proportional to the SOC. The higher the SOC, the more liquid is expelled. This is because the higher the SOC, the more intense the thermal runaway reaction, the higher the temperature, the more vigorous the gas generation, and the faster the expulsion rate, which results in a higher proportion of liquid material being ejected from the battery.

### 3.4. Gas Composition

**Figure 9:**
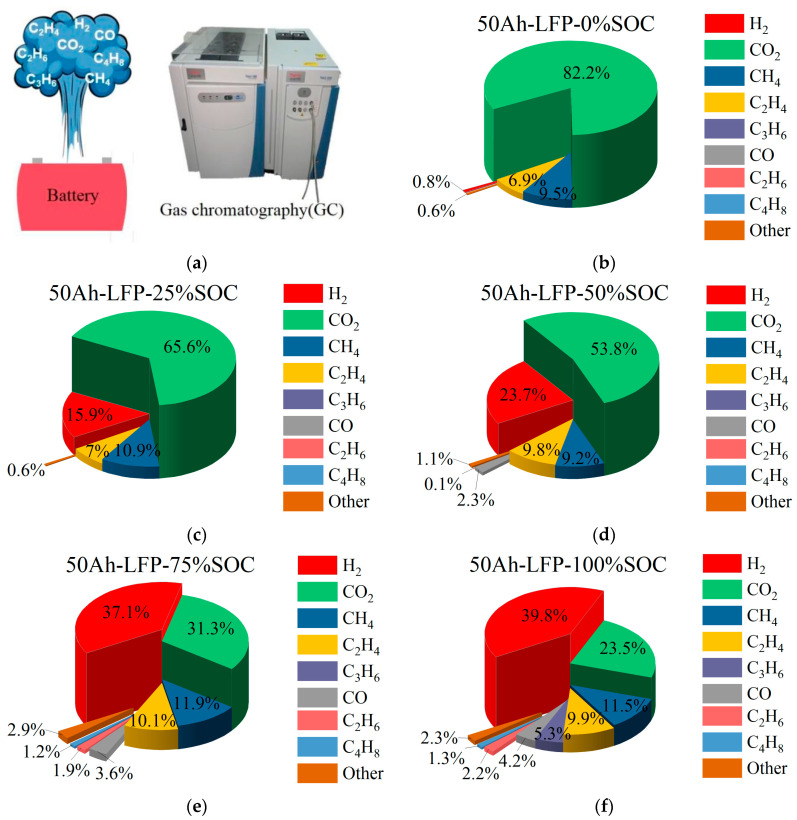
Gas composition of 50 Ah LiFePO_4_ battery triggered by stepwise heating to induce thermal runaway. (**a**) Thermal runaway gas analysis equipment. (**b**) Gas composition proportions of 0% SOC 50 Ah LiFePO_4_ battery during thermal runaway. (**c**) Gas composition proportions of 25% SOC 50 Ah LiFePO_4_ battery during thermal runaway. (**d**) Gas composition proportions of 50% SOC 50 Ah LiFePO_4_ battery during thermal runaway. (**e**) Gas composition proportions of 75% SOC 50 Ah LiFePO_4_ battery during thermal runaway. (**f**) Gas composition proportions of 100% SOC 50 Ah LiFePO_4_ battery during thermal runaway.

Figure 9a shows the gas products obtained after thermal runaway of LiFePO_4_ batteries at five different SOC levels, triggered by a 5 °C stepwise heating method. The thermal runaway gases were passed into a gas chromatograph (GC) to analyze the gas composition after thermal runaway. Figure 9b presents the gas composition of a 0% SOC 50 Ah LiFePO_4_ battery during thermal runaway. The gas with the highest proportion is CO_2_, which accounts for 82% of the total volume in the thermal runaway gas mixture, followed by CH_4_ (9.6%) and C_2_H_4_ (6.9%). The combined volume percentage of CO_2_, CH_4_, and C_2_H_4_ in the gas mixture is 98.6%.

Figure 9c shows the gas composition of a 25% SOC 50 Ah LiFePO_4_ battery during thermal runaway. CO_2_ remains the highest proportion gas in the mixture, accounting for 65.6%, with the second to fourth highest proportions being H_2_ (15.9%), CH_4_ (10.9%), and C_2_H_4_ (7%). The combined volume percentage of CO_2_, H_2_, CH_4_, and C_2_H_4_ in the gas mixture is 99.4%. Compared to the 0% SOC battery, the proportion of H_2_ has increased, indicating that as SOC increases, side reactions within the battery lead to changes in the gas composition.

Figure 9d shows the gas composition of a 50% SOC 50 Ah LiFePO_4_ battery during thermal runaway. The highest proportion gas is CO_2_, at 53.8%, followed by H_2_ (23.7%), CH_4_ (9.2%), and C_2_H_4_ (9.8%). The combined volume percentage of CO_2_, H_2_, CH_4_, and C_2_H_4_ in the gas mixture is 96.5%. This shows that as SOC increases further, the proportion of H_2_ continues to rise, while CO_2_’s proportion decreases significantly.

Figure 9e shows the gas composition of a 75% SOC 50 Ah LiFePO_4_ battery during thermal runaway. The highest proportion gas is H_2_, which accounts for 37.1%, followed by CO_2_ (31.3%), CH_4_ (11.9%), and C_2_H_4_ (10.1%). The combined volume percentage of CO_2_, H_2_, CH_4_, and C_2_H_4_ in the gas mixture is 90.4%. At this stage, the proportion of H_2_ exceeds that of CO_2_, indicating that the gas produced by side reactions during thermal runaway has increased significantly.

Figure 9f shows the gas composition of a 100% SOC 50 Ah LiFePO_4_ battery during thermal runaway. The highest proportion gas is H_2_, accounting for 39.8%, followed by CO_2_ (23.5%), CH_4_ (11.5%), C_2_H_4_ (9.9%), C_3_H_6_ (5.3%), and CO (4.2%). The combined volume percentage of CO_2_, H_2_, CH_4_, C_2_H_4_, C_3_H_6_, and CO in the gas mixture is 94.2%. At full charge, in addition to the gases from side reactions such as H_2_, CH_4_, and C_2_H_4_, other types of side reactions intensify, including the increased generation of gases like C_3_H_6_ and CO.

**Figure 10:**
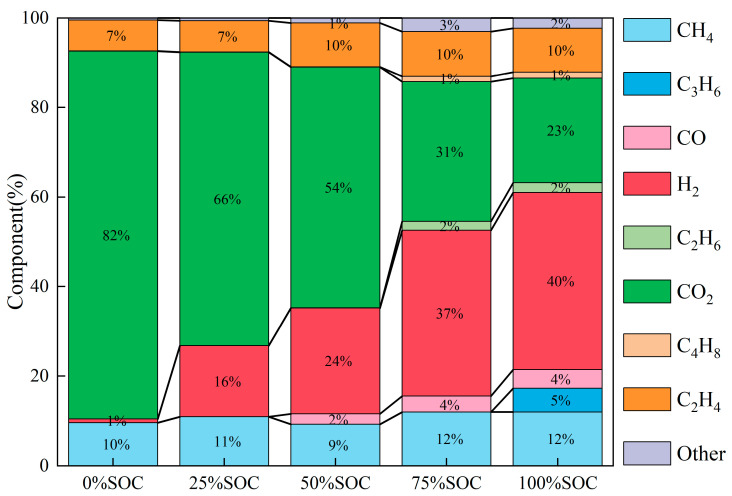
Volume proportions of gas products from thermal runaway of batteries at five SOC levels (rounded to one decimal place, volume fractions less than 1% not displayed).

The gas volume proportions of thermal runaway products at five SOC levels (0%, 25%, 50%, 75%, 100%) were normalized, with the volume fractions rounded to one decimal place. Volume fractions less than 1% are not displayed in Figure 10. The figure indicates that as the SOC increases, the proportion of H_2_ produced increases. This is because the LFP cathode material has an olivine crystal structure, which inherently possesses good thermal stability. However, at high SOC, the thermal runaway temperature is higher, leading to the decomposition of organic solvents and binders in the battery’s electrolyte, which generates large amounts of H_2_ [24,25,26].

The decomposition mechanism of organic solvents in the electrolyte upon heating is as follows:

(2) $$C_{3}H_{6}O_{3}\to CH_{3}OCOOCH+H_{2}↑$$

After thermal runaway, the graphite anode decomposes, and lithium reacts directly with the electrolyte and binder materials like polyvinylidene fluoride (PVDF) or carboxymethylcellulose (CMC), as follows:

(3) $$\text{–}CH_{2}\text{–}CF_{2}\text{–}+Li\to LiF+\text{–}CH=CF\text{–}+\frac{1}{2}H_{2}↑$$

(4) $$CMC\text{–}OH+Li\to CMC\text{–}OLi+\frac{1}{2}H2↑$$

As SOC increases, the proportion of CO_2_ produced decreases. This is because the thermal runaway temperature of high-SOC batteries is higher, leading to more types of side reactions inside the battery, and as a result, the proportion of CO_2_ produced decreases due to the increased generation of other gases.

The CO_2_ gas production mechanisms are as follows [27,28,29]:

Decomposition of the solid electrolyte interface (SEI) film:

(5) $$(CH_{2}OCO_{2}Li{)}_{2}\to {Li}_{2}CO_{3}+C_{2}H_{4}+CO_{2}↑+\frac{1}{2}O_{2}↑$$

Decomposition of the charging electrolyte interface (CEI) film:

(6) $${Li}_{2}CO_{3}+2HF\to 2LiF+CO_{2}↑+H_{2}O↑$$

Reaction of the battery anode with organic solvents:

(7) $$2Li+2C_{3}H_{4}O_{3}\to Li\text{–}O\text{–}(CH_{2}{)}_{4}\text{–}O\text{–}Li+2CO_{2}↑$$

Oxygen released from the cathode reacts with the electrolyte solvent:

(8) $$\frac{5}{2}O_{2}+C_{3}H_{4}O_{3}\to 3CO_{2}↑+H_{2}O↑$$

(9) $$4O_{2}+C_{4}H_{6}O_{3}\to 4CO_{2}↑+{3H}_{2}O↑$$

As SOC increases, the gas components such as C_2_H_4_ and CH_4_ remain largely unchanged.

### 3.5. Gas Production Volume

LiFePO_4_ batteries at five SOC levels were triggered into thermal runaway using the “HWS” stepwise heating method, and the environmental temperature and pressure data inside the sealed heating pressure chamber (SHPC) were measured before and after the experiment. Based on the ideal gas law (Equation (10)), the total gas production of four types of batteries was calculated [30].

(10) PV=nRT

(11) $$n=\frac{P_{2}V_{2}}{RT_{2}}-\frac{P_{1}V_{1}}{RT_{1}}$$

where

- n is the number of moles of gas produced (mol).
- P_2_ is the pressure inside the sealed heating pressure chamber (SHPC) after thermal runaway (Pa).
- V_2_ is the volume of the sealed heating pressure chamber (SHPC) (m^3^).
- R is the ideal gas constant (J/mol·K).
- T_2_ is the stable environment temperature inside the sealed heating pressure chamber (SHPC) after thermal runaway (K).
- P_1_ is the pressure inside the sealed heating pressure chamber (SHPC) before thermal runaway (Pa).
- V_1_ is the volume of gas inside the sealed heating pressure chamber (SHPC) before thermal runaway (m^3^).
- T_1_ is the environment temperature inside the sealed heating pressure chamber (SHPC) before the experiment (K).

**Figure 11:**
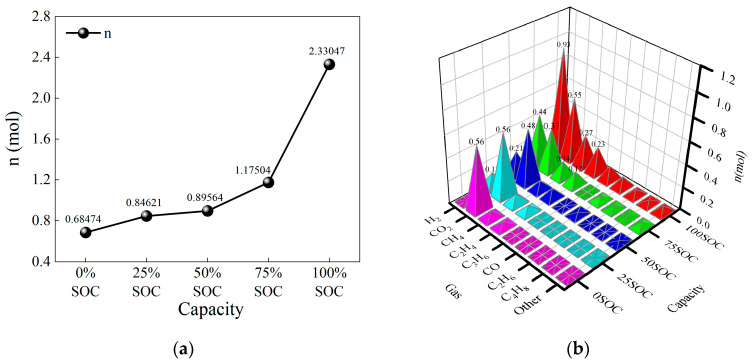
Amount of gas produced during thermal runaway of LiFePO_4_ batteries at different SOC levels. (**a**) Total amount of gas produced during thermal runaway of LiFePO_4_ batteries at different SOC levels. (**b**) Amount of different gases produced during thermal runaway of LiFePO_4_ batteries at different SOC levels.

Figure 11a shows the total amount of gas produced during thermal runaway for LiFePO_4_ batteries at different SOC levels. For 0% SOC, 25% SOC, and 50% SOC batteries, the total amount of gas produced was 0.68474 mol, 0.84621 mol, and 0.89564 mol, respectively, indicating that for SOC levels below 50%, the total amount of gas produced increases gradually. For a 75% SOC battery, the total amount of gas produced was 1.17504 mol, while for a 100% SOC battery, it was 2.33047 mol. This shows a sharp increase in gas production after 50% SOC, highlighting that as SOC increases, the number and intensity of side reactions in the battery lead to a significant rise in gas production.

Figure 11b presents the amounts of different gases produced during thermal runaway for LiFePO_4_ batteries at various SOC levels. It can be observed that as SOC increases, the production of CO_2_ remains relatively stable at different SOC levels: 0.56 mol (0% SOC), 0.56 mol (25% SOC), 0.48 mol (50% SOC), 0.37 mol (75% SOC), and 0.55 mol (100% SOC). This suggests that CO_2_ production is independent of SOC.

On the other hand, the amount of H_2_ produced increases significantly as SOC rises: 0.01 mol (0% SOC), 0.13 mol (25% SOC), 0.21 mol (50% SOC), 0.43 mol (75% SOC), and 0.93 mol (100% SOC). This trend indicates that as SOC increases, the thermal runaway temperature rises, and the reactions leading to hydrogen production from electrolyte decomposition and binder reactions become dominant among the side reactions.

As SOC increases, the amount of CH_4_ produced gradually increases: 0.07 mol (0% SOC), 0.09 mol (25% SOC), 0.08 mol (50% SOC), 0.14 mol (75% SOC), and 0.27 mol (100% SOC). Compared to H_2_, CH_4_ production increases more slowly, indicating that the side reactions generating CH_4_ are less intense, and the effect of the thermal runaway temperature on CH_4_ production is minimal.

Similarly, as SOC increases, the amount of C_2_H_4_ produced gradually rises: 0.05 mol (0% SOC), 0.06 mol (25% SOC), 0.09 mol (50% SOC), 0.12 mol (75% SOC), and 0.23 mol (100% SOC). The production of C_2_H_4_ increases more slowly, suggesting that the side reactions producing C_2_H_4_ are milder, and the thermal runaway temperature has a limited effect on its generation.

Other flammable gases show only a slight increase in production with SOC, with no significant variations.

### 3.6. Explosive Characteristics

**Figure 12:**
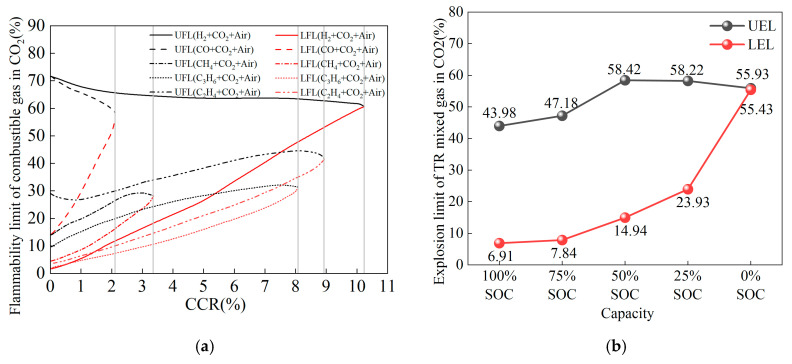
Flammability limits of single flammable gases in CO_2_ atmosphere and flammability limits of thermal runaway gases from LiFePO_4_ batteries at different SOC levels. (**a**) Variation trend of flammability limits for single flammable gases mixed with CO_2_ at different CCR ratios. (**b**) Flammability limits of thermal runaway gases from LiFePO_4_ batteries at different SOC levels considering the inerting effect of CO_2_.

Based on the measured gas compositions, there is a relatively high proportion of CO_2_ in the gas produced during the thermal runaway of LiFePO_4_ batteries. If we directly use Le Chatelier’s equation to calculate the flammability limits, this may lead to significant errors. Therefore, in this study, we mix CO_2_ with various flammable gases and treat the mixed gas as a single gas to redefine its flammability limits. Considering that gases such as C_2_H_6_ and C_4_H_8_ have proportions smaller than 2.3% during thermal runaway, the inerting effect of CO_2_ is not considered. As shown in Figure 12a, the flammability limits of a single flammable gas mixed with CO_2_ vary with the CCR (CO_2_ to flammable gas ratio). The CCR is calculated as follows:

(12) $$CCR=\frac{n_{{CO}_{2}}}{n_{i}}\times 100\%$$

where

- CCR is the dilution ratio of CO_2_ to the single flammable gas.
- n_CO2_ is the number of moles of CO_2_ (mol).
- n_i_ is the number of moles of a single gas (mol).

The thermal runaway gas compositions of the LiFePO_4_ battery at five different SOC levels are substituted into the above formula to determine the new flammability limits of the mixed gas (with CO_2_) [31]. These new flammability limits are then input into Le Chatelier’s equation (Equation (13)) to calculate the flammability limits as follows [32]:

(13) $$L_{mix}=(\sum_{i=1}^{n}\frac{x_{i}}{L_{i}}{)}^{-1}\times 100\%$$

- L_mix_ represents the flammability limits (both lower and upper) of the thermal runaway gases of the LiFePO_4_ battery.
- x_i_ is the mole percentage of the i-th gas in the thermal runaway gases of the LiFePO_4_ battery.
- L_i_ is the flammability limit of the i-th gas in the presence of CO_2_.

Using the above formulas, the flammability limits of the thermal runaway gases of LiFePO_4_ batteries at different SOC levels, considering the inerting effect of CO_2_, are shown in Figure 12b. Figure 12b demonstrates that the lower explosion limit (LEL) of the thermal runaway mixed gases from LiFePO_4_ batteries increases gradually as the SOC decreases above 50%. Below 50% SOC, the LEL rises sharply as the SOC decreases. Overall, the LEL of the thermal runaway mixed gases from LiFePO_4_ batteries increases with decreasing SOC, indicating that the thermal runaway mixed gases from batteries with low SOC have a higher LEL, making them less susceptible to ignition, whereas the thermal runaway mixed gases from batteries with high SOC have a lower LEL, thereby increasing the risk of combustion and explosion. This is because batteries with low SOC generate more CO_2_ during thermal runaway. In the combustible gas-CO_2_-air mixture, when the CO_2_ proportion is high, more oxygen is required to ignite a certain amount of fuel in order to ensure that the rate of destruction of the combustion chain reaction is lower than the rate of generation. As a result, both LEL and UEL are reduced.

The upper flammability limit (UEL) of the thermal runaway mixed gases of the LiFePO_4_ battery is 43.98% at 100% SOC, reaches its highest point of 58.42% at 50% SOC, and is 55.93% at 100% SOC. The UEL of the thermal runaway mixed gases increases with decreasing SOC, then slowly decreases. This suggests that for SOC levels below 50%, the UEL remains largely constant, fluctuating between 55.93% and 58.42%. For SOC levels above 50%, the UEL gradually decreases with increasing SOC. This is because at SOC levels below 50%, the volume proportion of CO_2_ in the thermal runaway gas is higher, and since CO_2_ is an inert gas, it dilutes and inertizes the flammable gases, leading to a higher UEL for batteries with SOC below 50%. Above 50% SOC, multiple side reactions occur, increasing the proportion of flammable gases and lowering the UEL of the thermal runaway mixed gases.

The flammability range of thermal runaway gases from LiFePO_4_ batteries is measured using the flammability range parameter H_F_, calculated using Equation (14) [33].

(14) $$H_{F}=UFL-LEL$$

Table 3 shows the variation of the flammability range parameter HFH_FHF for the thermal runaway mixed gases of the battery at different SOC levels. Between 25% SOC and 100% SOC, the flammability range fluctuates slightly, ranging from 34% to 43%. Within this SOC range, the flammability range of the battery’s thermal runaway mixed gases remains relatively stable. However, at 0% SOC, the flammability range of the thermal runaway mixed gases is 0.5, indicating that in the absence of charge, the flammability range of the battery’s thermal runaway mixed gases is very small, and its flammability significantly decreases.

Based on the above analysis, after thermal runaway, the flammable gas mixture generated by the battery’s thermal runaway can be diluted and inerted by rapidly releasing CO_2_ gas, thereby quickly reducing the flammable range of HF and achieving the goal of suppressing the combustion and explosion of the thermal runaway gas mixture.

## 4. Conclusions

This study used a sealed heating pressure chamber (SHPC) to trigger the thermal runaway of a 50 Ah LiFePO_4_ battery via stepwise heating (“HWS”), with five different SOC levels including 0%, 25%, 50%, 75%, and 100% SOC. The temperature, voltage, and chamber pressure during the thermal runaway process were monitored, and the gas composition of the mixed gases post-thermal runaway was analyzed using GC equipment. The following conclusions were obtained:

1. The valve opening temperature T_1_ during battery thermal runaway remained in the range of 156.7 °C to 159.9 °C across different SOC levels, with minimal fluctuation. This indicates no significant correlation between T_1_ and SOC. The rapid self-heating onset temperature T_2_ during thermal runaway was between 180 °C and 186 °C across all SOCs, with little temperature variation, suggesting no significant correlation between T_2_ and SOC. The maximum temperature T_3_ during thermal runaway was positively correlated with SOC, meaning that as SOC increases, T_3_ increases, reaching a maximum of 357.4 °C. A higher SOC leads to a larger internal short-circuit current, higher temperatures, and more intense thermal runaway reactions and eruptions. The side reactions between the anode and cathode materials and the electrolyte generate more gas and electrolyte vapor, which are expelled in greater quantities, resulting in an increased mass loss rate with the increase in SOC. The experiments show that the mass loss rate during thermal runaway triggered by stepwise heating in a 50 Ah LiFePO_4_ battery is around 14% to 21%.
2. For the 50 Ah LiFePO_4_ battery at five SOC levels, the battery casing experienced similar deformations but did not suffer any rupture or melting. This is because the maximum temperature during thermal runaway was only 357.4 °C, which did not reach the aluminum shell melting point of 660 °C, thus maintaining the integrity of the battery casing. The valve opening temperature T_1_ was around 158 °C (ranging from 156.7 °C to 159.9 °C), suggesting that the critical point of the battery safety valve is largely unaffected by SOC and is mainly influenced by temperature.
3. The volume percentage of gases produced during thermal runaway at five different SOC levels (0%, 25%, 50%, 75%, 100%) was normalized, revealing that as SOC increased, the proportion of H_2_ produced increased, while the proportion of CO_2_ decreased. This is because the thermal runaway temperature of higher SOC batteries is higher, leading to more types of side reactions inside the battery, which results in an increase in other types of gases and a decrease in the proportion of CO_2_ produced.
4. Below 50% SOC, the total moles of gas produced during thermal runaway were 0.68474 mol (0% SOC), 0.84621 mol (25% SOC), and 0.89564 mol (50% SOC), showing a slow increase in gas production. For SOC levels above 50%, the total moles of gas produced were 1.17504 mol (75% SOC) and 2.33047 mol (100% SOC). As SOC increased, the number and types of side reactions during thermal runaway increased, causing a sharp rise in gas production.
5. As SOC increased, the amount of CO_2_ produced varied little across different SOCs, indicating that the side reaction producing CO_2_ is independent of SOC. However, the amount of H_2_ produced increased sharply: 0.01 mol (0% SOC), 0.13 mol (25% SOC), 0.21 mol (50% SOC), 0.43 mol (75% SOC), and 0.93 mol (100% SOC). As the thermal runaway temperature increased, hydrogen production from electrolyte decomposition and binder reactions became dominant among the side reactions. The amounts of CH_4_ and C_2_H_4_ produced gradually increased, suggesting that the side reactions producing these gases were relatively mild, with minimal influence from thermal runaway temperature. Other flammable gases showed little change in production with varying SOC.
6. Considering the inerting effect of CO_2_ in the thermal runaway gas, the recalculated lower flammability limit (LEL) of the thermal runaway mixed gas for the LiFePO_4_ battery was 6.91% at 100% SOC and 55.43% at 0% SOC. The LEL increased as SOC decreased, indicating that the thermal runaway mixed gas of low SOC batteries has a higher LEL and is harder to ignite, while the thermal runaway mixed gas of high SOC batteries has a lower LEL, making it more susceptible to explosion risks. The flammability range parameter H_F_ for the mixed gas remained relatively stable between 34% and 43% in the SOC range of 25% to 100%, while at 0% SOC, the flammability range was minimal, and the flammability sharply decreased.

Based on the above conclusions, future research can focus on thermal management strategies to suppress thermal runaway in lithium iron phosphate batteries. For example, before thermal runaway occurs, liquid cooling methods can be used to control the temperature of individual cells and prevent them from reaching the critical thermal runaway temperature. For batteries that have already begun self-heating, strategies such as rapid external discharge to reduce the SOC can be employed to interrupt or mitigate thermal runaway. For batteries that have already undergone thermal runaway, injecting CO_2_ gas can help inert the flammable gas mixture generated, thereby reducing the risk of combustion and explosion.

## Figures and Tables

**Table 1:** Cell performance.

Parameter	Specification	Condition
Nominal Capacity	50 Ah	25 °C, 0.5 C
Minimum Discharge Energy	160 Wh	25 °C, 0.5 C
Operating Voltage Range	2.5~3.65 V	>0 °C
Cathode	LiFePO_4_	N.A.
Anode	Graphite	N.A.
Standard Voltage	3.20 V	25 °C, 0.5 C
Charge Operation Temperature Range	−10~55 °C	N.A.
Discharge Operation Temperature Range	−30~60 °C	N.A.
Alternating Current Resistance	0.2~0.6 mΩ	25 °C, BOL, 17% SOC, 1 kHz.
Weight	1130 ± 50 g	N.A.
Thickness	39.7 ± 0.3 mm	N.A.
Length	147.9 ± 0.8 mm	N.A.
Height	95.4 ± 0.5 mm	N.A.
Material	Aluminum alloy	N.A.
Cathode Current Collector	Aluminum foil	N.A.
Anode Current Collector	Copper foil	N.A.
Electrolyte Type	Liquid Electrolyte	N.A.
Electrolyte Composition	Carbonate + Lithium hexafluorophosphate	N.A.
Cycle Life	≥6000	Under 150 kgf initial compression force, 25 °C 0.5 C/0.5 C, 2.5 V~3.65 V, cycle to 80%SOH

**Table 2:** Mass loss before and after battery thermal runaway.

Capacity	0% SOC	25% SOC	50% SOC	75% SOC	100% SOC
Initial Mass m_0_ (g)	1130	1130	1130	1130	1130
Post-experiment Mass m_1_ (g)	970	948	936.2	909.4	891.6
Mass Loss Δm (g)	160	182	193.8	220.6	238.4
Mass Loss Rate φ_loss_ (%)	14.159292	16.1061947	17.1504425	19.5221239	21.0973451

**Table 3:** Variation of the flammability range parameter HFH_FHF of battery thermal runaway mixed gases at different SOC levels.

Capacity	100% SOC	75% SOC	50% SOC	25% SOC	0% SOC
UEL	43.98	47.18	58.42	58.22	55.93
LEL	6.91	7.84	14.94	23.93	55.43
H_F_	37.07	39.34	43.48	34.29	0.5

## Data Availability

The original contributions presented in the study are included in the article, further inquiries can be directed to the corresponding author.

## References

[1] Chavan S., Venkateswarlu B., Prabakaran R., Salman M., Joo S.W., Choi G.S., Kim S.C. (2023). Thermal runaway and mitigation strategies for electric vehicle lithium-ion batteries using battery cooling approach: A review of the current status and challenges. J. Energy Storage.

[2] Hu G., Huang P., Bai Z., Wang Q., Qi K. (2021). Comprehensively analysis the failure evolution and safety evaluation of automotive lithium ion battery. ETransportation.

[3] Wu H., Chen S., Hong Y., Xu C., Zheng Y., Jin C., Chen K., He Y., Feng X., Wei X. (2024). Thermal safety boundary of lithium-ion battery at different state of charge. J. Energy Chem..

[4] Huang P., Yao C., Mao B., Wang Q., Sun J., Bai Z. (2020). The critical characteristics and transition process of lithium-ion battery thermal runaway. Energy.

[5] Xiao Y., Yang F., Gao Z., Liu M., Wang J., Kou Z., Lin Y., Li Y., Gao L., Chen Y. (2023). Review of mechanical abuse related thermal runaway models of lithium-ion batteries at different scales. J. Energy Storage.

[6] Li H., Zhou D., Zhang M., Liu B., Zhang C. (2023). Multi-field interpretation of internal short circuit and thermal runaway behavior for lithium-ion batteries under mechanical abuse. Energy.

[7] Wei D., Zhang M., Zhu L., Chen H., Huang W., Yao J., Yuan Z., Xu C., Feng X. (2022). Study on thermal runaway behavior of Li-ion batteries using different abuse methods. Batteries.

[8] Wang Y., Feng X., Huang W., He X., Wang L., Ouyang M. (2023). Challenges and opportunities to mitigate the catastrophic thermal runaway of high-energy batteries. Adv. Energy Mater..

[9] Kong D., Lv H., Ping P., Wang G. (2023). A review of early warning methods of thermal runaway of lithium ion batteries. J. Energy Storage.

[10] Ren D., Feng X., Liu L., Hsu H., Lu L., Wang L., He X., Ouyang M. (2021). Investigating the relationship between internal short circuit and thermal runaway of lithium-ion batteries under thermal abuse condition. Energy Storage Mater..

[11] Huang S., Du Z., Zhou Q., Snyder K., Liu S., Zhang G. (2021). In situ measurement of temperature distributions in a Li-ion cell during internal short circuit and thermal runaway. J. Electrochem. Soc..

[12] Hong J., Wang Z., Qu C., Zhou Y., Shan T., Zhang J., Hou Y. (2022). Investigation on overcharge-caused thermal runaway of lithium-ion batteries in real-world electric vehicles. Appl. Energy.

[13] Liu J., Wang Z., Bai J. (2022). Influences of multi factors on thermal runaway induced by overcharging of lithium-ion battery. J. Energy Chem..

[14] Zhang Q., Niu J., Zhao Z., Wang Q. (2022). Research on the effect of thermal runaway gas components and explosion limits of lithium-ion batteries under different charge states. J. Energy Storage.

[15] Wang Z., Zhu L., Liu J., Wang J., Yan W. (2022). Gas Sensing Technology for the Detection and Early Warning of Battery Thermal Runaway: A Review. Energy Fuels.

[16] Qiu M., Liu J., Cong B., Cui Y. (2023). Research Progress in Thermal Runaway Vent Gas Characteristics of Li-Ion Battery. Batteries.

[17] Wei G., Huang R., Zhang G., Jiang B., Zhu J., Guo Y., Han G., Wei X., Dai H. (2023). A comprehensive insight into the thermal runaway issues in the view of lithium-ion battery intrinsic safety performance and venting gas explosion hazards. Appl. Energy.

[18] Willstrand O., Pushp M., Andersson P., Brandell D. (2023). Impact of different Li-ion cell test conditions on thermal runaway characteristics and gas release measurements. J. Energy Storage.

[19] Zhang Q., Liu T., Hao C., Qu Y., Niu J., Wang Q., Chen D. (2022). In situ Raman investigation on gas components and explosion risk of thermal runaway emission from lithium-ion battery. J. Energy Storage.

[20] Qi C., Liu Z., Lin C., Liu X., Liu D., Li Z., Yi A. (2024). The gas production characteristics and catastrophic hazards evaluation of thermal runaway for LiNi0. 5Co0. 2Mn0. 3O2 lithium-ion batteries under different SOCs. J. Energy Storage.

[21] Cui Y., Shi D., Wang Z., Mou L., Ou M., Fan T., Bi S., Zhang X., Yu Z., Fang Y. (2023). Thermal Runaway Early Warning and Risk Estimation Based on Gas Production Characteristics of Different Types of Lithium-Ion Batteries. Batteries.

[22] Feng X., Zheng S., Ren D., He X., Wang L., Liu X., Li M., Ouyang M. (2019). Key Characteristics for Thermal Runaway of Li-ion Batteries. Energy Procedia.

[23] Doose S., Hahn A., Fischer S., Müller J., Haselrieder W., Kwade A. (2023). Comparison of the consequences of state of charge and state of health on the thermal runaway behavior of lithium ion batteries. J. Energy Storage.

[24] Chen J., Xu C., Wang Q., Wang H., Peng Y., Liu J., Zhang J., Zhang G., Lu L., Feng X. (2025). The thermal-gas coupling mechanism of lithium iron phosphate batteries during thermal runaway. J. Power Sources.

[25] Jia Z., Qin P., Li Z., Wei Z., Jin K., Jiang L., Wang Q. (2022). Analysis of gas release during the process of thermal runaway of lithium-ion batteries with three different cathode materials. J. Energy Storage.

[26] Yang M., Rong M., Ye Y., Yang A., Chu J., Yuan H., Wang X. (2023). Comprehensive analysis of gas production for commercial LiFePO4 batteries during overcharge-thermal runaway. J. Energy Storage.

[27] Bugryniec P.J., Resendiz E.G., Nwophoke S.M., Khanna S., James C., Brown S.F. (2024). Review of gas emissions from lithium-ion battery thermal runaway failure—Considering toxic and flammable compounds. J. Energy Storage.

[28] Wang S., Song L., Li C., Tian J., Jin K., Duan Q., Wang Q. (2023). Experimental study of gas production and flame behavior induced by the thermal runaway of 280 Ah lithium iron phosphate battery. J. Energy Storage.

[29] Su L., Yang F., Hu W., Chen S., Lyu N. (2024). Thermal Runaway Gas Generation of Lithium Iron Phosphate Batteries Triggered by Various Abusive Conditions. J. Energy Eng..

[30] Lin C., Yan H., Qi C., Mao J., Lao L., Sun Y., Ma T., Liu D. (2024). Research on thermal runaway and gas generation characteristics of NCM811 high energy density lithium-ion batteries under different triggering methods. Case Stud. Therm. Eng..

[31] Kondo S., Takizawa K., Takahashi A., Tokuhashi K. (2006). Extended Le Chatelier’s formula for carbon dioxide dilution effect on flammability limits. J. Hazard. Mater..

[32] Liu Z.-K., Ågren J., Hillert M. (1996). Application of the Le Chatelier principle on gas reactions. Fluid Phase Equilibria.

[33] Li W., Wang H., Zhang Y., Ouyang M. (2019). Flammability characteristics of the battery vent gas: A case of NCA and LFP lithium-ion batteries during external heating abuse. J. Energy Storage.

